# Determining the appropriate soybean meal inclusion level in lactation diets for sows endemically infected with porcine reproductive and respiratory syndrome virus (PRRSv)

**DOI:** 10.1093/tas/txaf054

**Published:** 2025-05-04

**Authors:** Danielle C Johnson, Dustin D Boler, Jeremy G Perez, Oscar M Medina, Jorge Estrada, Deanne Corzatt, Kelsey L Kyle, Eric Parr, Casey Neill, Aaron M Gaines, Michael W Welch

**Affiliations:** Carthage Veterinary Service Ltd., Carthage, IL 62321, USA; Carthage Veterinary Service Ltd., Carthage, IL 62321, USA; Carthage Veterinary Service Ltd., Carthage, IL 62321, USA; Carthage Veterinary Service Ltd., Carthage, IL 62321, USA; Carthage Veterinary Service Ltd., Carthage, IL 62321, USA; Carthage Veterinary Service Ltd., Carthage, IL 62321, USA; Carthage Veterinary Service Ltd., Carthage, IL 62321, USA; Carthage Veterinary Service Ltd., Carthage, IL 62321, USA; Carthage Veterinary Service Ltd., Carthage, IL 62321, USA; Ani-Tek Group, LLC, Shelbina, MO 63468, USA; Carthage Veterinary Service Ltd., Carthage, IL 62321, USA

**Keywords:** farrowing performance, lactation, pig, porcine reproductive and respiratory syndrome virus, PRRSv

## Abstract

Soybean meal (SBM) contains many bioactive compounds, such as isoflavones, which possess anti-inflammatory and anti-oxidative properties that may provide nutritional intervention to pigs infected with porcine reproductive and respiratory syndrome virus (PRRSv). The disease results in abortions, stillborn piglets, and overall impairs reproductive success in sows. Today, there are no data available on feeding SBM to sows infected with PRRSv to mitigate the negative impacts of PRRSv on sow and litter performance. A total of 960 sows were used for this study at an endemically PRRSv-infected farm. There were 4 dietary treatments with 20%, 25%, 30%, or 35% SBM inclusion in the lactation diet. All dietary treatments were formulated to target 65 g of standardized ileal digestibility (SID) Lys and 24.1 Mcal of metabolizable energy (ME) intake per day regardless of SBM inclusion. Daily feed intakes of sows were recorded prior to farrowing and throughout lactation. The total number of pigs born, pigs born alive, stillbirths, and mummies were recorded for each litter within 24h of farrowing. The individual body weight of each piglet was collected at birth and again at weaning. Pig mortalities were recorded for the entire lactation period. Body condition score (BCS) of sows at entry into farrowing room was not different (*P* = 0.32) among treatments, however BCS at weaning was at least 0.09 units greater (*P* ≤ 0.03) in sows fed 20% SBM compared to all other treatments. Sows fed 20% SBM in the diet consumed at least 1.74 g/d more (*P* ≤ 0.05) SID lysine and 0.57 Mcal/kg more (*P* ≤ 0.05) metabolizable energy compared to all other treatments. The number and percentage of pigs born alive were not different (*P* ≥ 0.37) among treatments. The number of pigs weaned was not different (*P* = 0.71) among treatments. The coefficient of variation (CV) of the weight of total pigs born per litter was not different (*P* = 0.54) among treatments. Average starting weight, average weaning weight, and weaning weights CV were not different (*P* ≥ 0.19) among treatments. Average daily piglet gain was not different (*P* = 0.49) among treatments. Feed efficiency improved with higher SBM inclusion because of reduced feed intake, which significantly reduced BCS at weaning. As a result, sows fed the lowest SBM level (20%) ate more, maintained better BCS, and may be better positioned for future performance and longevity.

## Teaser

Soybean meal (SBM) contains relatively high levels of isoflavones which possess anti-inflammatory and anti-oxidative properties. This makes SBM a feed ingredient that may provide nutritional intervention to pigs infected with porcine reproductive and respiratory syndrome virus (PRRSv), potentially mitigating the reductions in litter growth and performance. This trial used 4 dietary treatments with increasing SBM inclusions in the lactation diets of multiparous sows. Those SBM levels were 20%, 25%, 30%, or 35%. All diets were formulated to include a target of 65 g of standardized ileal digestibility (SID) Lys and 24.1 Mcal/kg of metabolizable energy (ME) intake per day regardless of SBM inclusion. Increasing the inclusion rate of SBM in the diet of sows endemically infected with PRRSv improved lactation feed efficiency but did not significantly reduce piglet mortality or improve litter performance. However, sows fed the lowest SBM inclusion rate (20%) had greater feed intake and improved body condition at weaning which may position them to have improved subsequent performance and longevity within the herd.

## INTRODUCTION

Porcine reproductive and respiratory syndrome virus (PRRSv) is a disease that results in abortions, stillborn piglets, and overall impairs reproductive success in sows (Tousignant et al., 2015). Growth performance in pre-weaned and weaned pigs is also detrimentally impacted by PRRSv (Boyd et al., 2010; Rochell et al., 2015; Quezada-Fraide et al., 2021). The damaging effects of PRRSv on both breeding and growing herds result in a 1.2 billion USD annual revenue loss (Osemeke et al., 2024), making it one of the most economically impactful diseases in the swine industry.

Eradicating PRRSv and keeping the disease from spreading is managed through implementation of biosecurity and vaccination protocols, but these strategies have only been marginally successful (Nan et al,. 2017; Havas et al., 2023) Therefore, nutritional intervention is often the focus for alleviating the effects of PPRSv outbreaks on commercial sow farms. Soybean meal (SBM) is the most commonly used protein source in swine diets in the United States with 74% of total soybean meal produced being used to feed poultry and swine (United Soybean Board, 2023). In addition to being a good source of crude protein and energy, SBM is comprised of relatively high levels of bioactive compounds such as isoflavones, saponins, and select carbohydrates. These are thought to possess anti-inflammatory and anti-oxidative properties (Smith and Dilger, 2018; White et al., 2024) and may improve growth performance and mitigate the effects of illness in pigs (Boyd et al., 2010; Rochell et al., 2015).

Feeding SBM to pigs infected with PRRSv has been investigated heavily in nursery (Rocha et al., 2013; Rochell et al., 2015; Smith et al., 2019) and grow-finish pigs (Boyd et al., 2010; Smith et al., 2020). The evidence supporting feeding increased levels of SBM to growing finishing pigs during a PRRSV challenge is inconsistent. This is likely because experimental PRRS challenge trials and endemic challenge trials can be difficult to replicate due to variation in environmental factors, severity of strains, and impacts of coinfections with other diseases. Most PRRSv research has investigated PRRSv impact where pigs were experimentally inoculated using a small number of pigs in a controlled environment rather than natural infection at a commercial farm. No producer would purposely infect a sow herd with PRRSv, and natural outbreaks are never planned. Therefore, there are no data available on the effects of increasing SBM in lactation diets to potentially mitigate the impact of PRRSv on lactating sow or nursing pig performance. This study took advantage of an opportunity to feed 4 levels of SBM to sows on an endemically PRRSv-infected commercial sow farm in Western Illinois. The hypothesis was that as SBM inclusion increased in the lactation diet, sow and litter performance would improve.

## MATERIALS AND METHODS

All experimental procedures were reviewed and approved by the Carthage Veterinary Services IACUC committee (protocol #2024-29).

### Timeline

**Figure 1.**
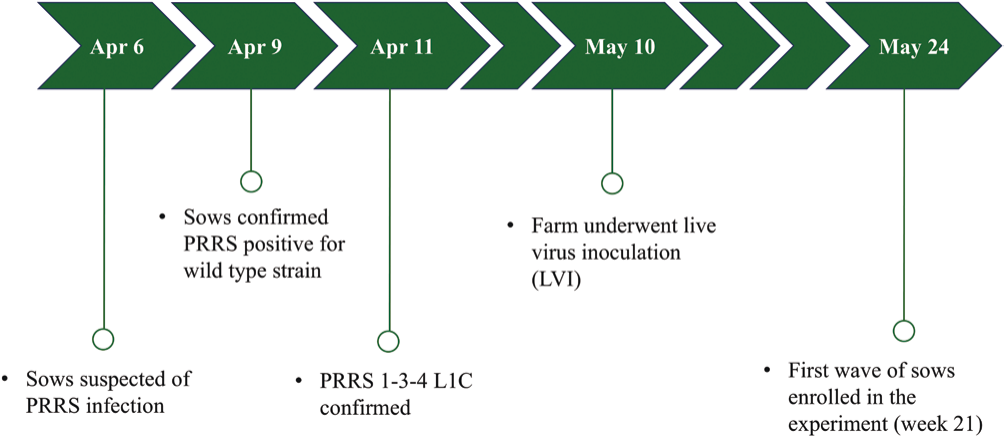
Experimental timeline of events prior to the start of the study.

**Figure 2.**
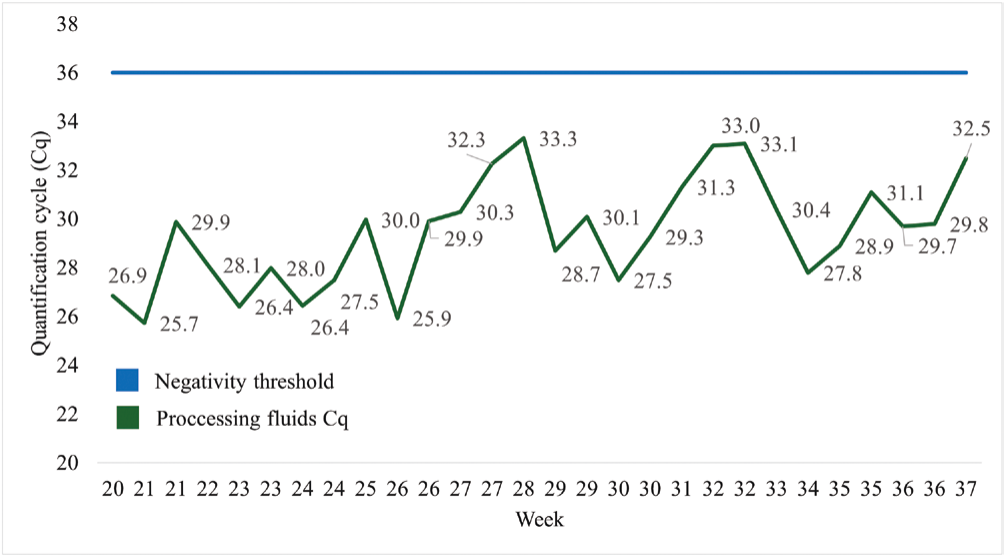
Weekly quantification cycles (Cq) of processing fluids from pigs nursing sows on trial during the 18-wk experimental period. A quantification cycle threshold of > 36 was considered negative for porcine reproductive and respiratory virus (PRRSv). All processing fluids had Cq values < 36 for the entirety of the trial

Sows were suspected of PRRSv infection on April 6, 2024 (Figure 1). Sows were confirmed PRRSv positive, and the strain was confirmed as 1-3-4 L1C within the next 5 d. The farm underwent live virus inoculation (LVI) a month later (May 10, 2024) and the first wave (block) of sows was enrolled in the trial 14 d later (May 24, 2024). All piglet processing fluids had Cq values < 36 for the entire experimental period (Figure 2), implying sows and piglets were positive for the PRRSv virus during the entirety of the trial.

### Animals and Housing

A total of 960 sows (PIC 1050 Camborough) were fed in the summer of 2024 at a commercial sow farm near Carthage, Illinois. Sows that had completed 8 parities or more were not enrolled in the study. Sows were moved from gestation pens into farrowing rooms at a target of 112 d of gestation. Sows were weighed, measured with a caliper at the last rib (scale 1 to 20; < 8 considered thin, > 11 considered over-conditioned (Knauer and Baitinger, 2015), and visually assigned a body condition score (BCS) upon entering the farrowing room (measured on a scale from 0 to 5). Each farrowing crate (1.5 m × 2.1 m total, with 0.6 m × 2.1 m for sows) had one nipple waterer, a feeder for the sow, and a heat lamp for the pigs.

### Processing Fluids and PCR

Processing fluids were obtained through standard operating procedures (SOP) for castration and tail docking of piglets during normal processing procedures. Testicles and tails from piglets of sows on trial were collected in Ziploc bags. The liquid accumulated in the bottom of the bag was decanted into a 50ml tube before being transported to Carthage Veterinary Diagnostics Laboratory and evaluated for PRRSv. Processing fluids over multiple farrowing rooms and multiple days were pooled into 1 weekly sample. Samples were tested by RT-qPCR, and the results were reported as quantification cycle (Cq) values. A cycle quantification threshold value of < 36 was considered positive for PRRSv as determined by Iowa State University Diagnostic Laboratory standard operating procedures.

### Diets, Feeding, and Experimental Design

All diets were formulated (Table 1) to meet or exceed the current National Research Council’s (2012) guidelines and PIC’s recommendations for lactating sows. Diets were manufactured at a commercial feed mill in Carthage, IL. Diets were formulated to provide 65 g of standardized ileal digestibility (SID) Lys and 24.1 Mcal of metabolizable energy (ME) regardless of SBM inclusion to sows that consume 7.30 kg (Estrada et al., 2024a) of complete feed per day. Additionally, the following modified ME values were used for diet formulation: Corn (3,340 kcal/kg), SBM (3,263 kcal/kg), Fat Vegetable Oil (8,578 kcal/kg), L-Lysine HCl 78% (4,350 kcal/kg), L-Threonine 99% (3,776 kcal/kg), L-Valine 98% (5,580 kcal/kg), DL-Methionine 99% (5,353 kcal/kg), L-Isoleucine 98.5% (6,151 kcal/kg), L-Tryptophan 98.5% (6,164 kcal/kg). (Table 1, Table 2). In anticipation of reductions in ADFI (Pedro Mil-Homens et al., 2024), diets were formulated to include a greater concentration of total lysine relative to previous lactation diets (Estrada et al., 2024a) fed at that farm. Sows were fed a standard gestation diet the day they were moved into the farrowing room and started on their respective research diet the following day, which continued until weaning.

**Table 1.** Diet formulation of experimental lactation diets

Ingredient, %	20% Soybean meal	25% Soybean meal	30% Soybean meal	35% Soybean meal
Corn	74.07	69.36	64.64	59.93
SBM	20.00	25.00	30.00	35.00
Fat Vegetable Oil	1.50	1.64	1.77	1.90
Calcium Carbonate	1.36	1.36	1.35	1.35
Monocal Phosphate 21%	0.97	0.89	0.82	0.75
Salt	0.57	0.57	0.56	0.56
L-Lysine HCl 78%	0.51	0.36	0.21	0.06
L-Threonine 99%	0.22	0.15	0.08	0.02
DL-Methionine 99%	0.12	0.08	0.04	-
L-Tryptophan 98.5%	0.05	0.03	0.02	-
L-Valine 98%	0.12	0.08	0.04	-
L-Isoleucine 98.5%	0.07	0.04	0.02	-
Choline Chloride 60%	0.10	0.10	0.10	0.10
VTM—Carthage Sow 5 w/Phytase	0.25	0.25	0.25	0.25
Microgrits BLUE color	0.10	-	-	-
Microgrits RED color	-	0.10	-	-
Microgrits GREEN color	-	-	0.10	-
Microgrits ORANGE color	-	-	-	0.10
Total	100.00	100.00	100.00	100.00

**Table 2.** Nutrient composition of experimental lactation diets (as-fed basis)

	20% Soybean meal		25% Soybean meal		30% Soybean meal		35% Soybean meal	
Composition^1^^,^^2^	Calc.	Analy.	Calc.	Analy.	Calc.	Analy.	Calc.	Analy.
Mod ME, kcal/kg	3308		3308		3308		3308	
Dry Matter	86.80	86.43	86.84	86.56	86.88	87.00	86.92	86.81
Crude Protein, %	15.86	16.78	17.56	17.90	19.25	19.65	20.95	21.55
Crude Fat, %	4.11	3.96	4.16	4.14	4.21	4.16	4.26	4.13
Ash, %	5.05	4.39	5.22	4.44	5.39	4.53	5.56	4.81
NDF, %	7.23	6.83	7.23	7.30	7.23	6.93	7.22	7.25
Analyzed Ca, %	0.77	0.74	0.77	0.74	0.77	0.77	0.77	0.79
Phosphorus, %	0.52	0.51	0.53	0.54	0.53	0.53	0.54	0.57
Ca:P	1.49	1.44	1.47	1.36	1.45	1.44	1.43	1.40
STTD Phosphorus, %	0.43		0.43		0.43		0.43	
Phytase, FTU/kg	750		750		750		750	
Sodium, %	0.24	0.19	0.24	0.19	0.24	0.18	0.24	0.20
Iron, ppm	221	280	219	279	219	288	217	285
Manganese, ppm	62	83	63	87	63	77	64	86
Copper, ppm	20	29	21	28	22	27	22	30
Zinc, ppm	152	147	154	151	156	126	158	156
Lysine, %	1.14		1.15		1.16		1.18	
SID Lysine, %	1.03		1.03		1.03		1.03	
SID Lys:Cal ME ratio	3.15		3.15		3.14		3.14	
SID Lys:Cal NE ratio	3.95		3.95		3.95		3.95	
SID AA:SID Lys (ratio)								
Iso:Lys	0.56		0.62		0.67		0.73	
Leu:Lys	1.13		1.24		1.35		1.46	
Met + Cys:Lys	0.53		0.53		0.54		0.54	
Thr:Lys	0.64		0.64		0.64		0.64	
Trp:Lys	0.19		0.20		0.21		0.22	
Val:Lys	0.67		0.71		0.75		0.78	
Soy isoflavones								
Daidzein^3^, ppm		<10.00 to 13.10		<10.00 to 13.05		<10.00 to 14.15		<10.00 to 16.30
Daidzin^3^, ppm		275.25		327.75		410.25		460.50
Genistein^3^, ppm		<10.00		<10.00		<10.00		<10.00
Genistin^3^, ppm		370.00		433.75		529.00		596.25
Glycitein^3^, ppm		18.65		17.08		17.08		18.05
Glycitin^3^, ppm		54.33		56.48		78.68		82.78
Total as Glucosides, ppm		739.50		855.75		1056.50		1165.00

^1^Diets were in meal form and manufactured at the NSI feed mill (Carthage, IL).

^2^Analyses were carried out by Midwest Labs (Omaha, NE) using wet chemistry.

^3^Isoflavone analyses were performed by Eurofins Microbiology Laboratories, Madison, WI.

Sows were initially blocked by parity (1, 2, 3, and 4+) and randomly allotted to 1 of 4 treatments with a SBM inclusion of 20%, 25%, 30%, and 35%. Overall, 209 sows were fed treatment 1 (20% SBM), 219 fed treatment 2 (25% SBM), 222 fed treatment 3 (30% SBM), and 227 fed treatment 4 (35% SBM). Sows were assigned to treatment diets when they entered the farrowing room based on breed date and parity. Prior to farrowing, sows were fed 2.27 kg of their respective lactation treatment diet in a single meal at 0700 hours using a calibrated volumetric Sow Max hopper (Hog Slat Inc., Newton Grove, NC). Sows were provided ad libitum access to their respective treatment diet immediately after the first piglet was observed. Feed was added to the feeders, and feed refusals were recorded daily. Feed remaining in the feeders on the day of farrowing and after weaning was measured to calculate individual feed disappearance from loading until farrowing, farrowing until weaning, and overall feed intake.

Research diets were manufactured at a commercial toll mill. Reasonable variations in formulated and analyzed nutrient values were expected. A weekly sample of each lactation diet was collected and held until the end of the trial. A composite sample of each treatment was sent to a commercial laboratory (Midwest Laboratories, Omaha, NE) and analyzed in duplicate for crude protein (AOAC 990.03, 2006), crude fat (AOAC 2003.05, 2007), neutral detergent fiber (Ankom Technology/AOAC 2001.11, 2005), ash (AOAC 942.05, 2012), calcium, phosphorous, sodium, iron, manganese, copper, and zinc (AOAC 985.01, 1996). Additionally, composite samples of each treatment were sent to another commercial laboratory (Eurofins Microbiology Laboratories, Madison, WI) and analyzed in duplicate for soy isoflavones (Hsieh et al., 2004).

### Farrowing and Data Collection

Sows were monitored during farrowing every 25 to 30 min between 0645 and 1500 hours to record when the first pig was born. Sows that farrowed overnight were checked the following morning. Sows that had not yet farrowed by d 116 of gestation were induced with PGF2α (2c.c., Lutalyse, Zoetis Inc., Kalamazoo, MI). Within 24 hours of farrowing, numbers of total pigs born, total live pigs born, stillbirths, and mummies were recorded for each litter. Individual pig birth weights were recorded using radio frequency identification tags (RFID; LeeO, Prairie Systems, Spencer, IA) and used to calculate a litter birth weight. Weights of stillbirth pigs and mummies were weighed and added to the litter birth weight to calculate a litter average pig birth weight. Empty sow weight after farrowing was estimated by subtracting conceptus weight from pre-farrow adjusted weight. Conceptus weight was estimated using the equation: 0.137 + 1.329 × total pigs born × litter average pig birth weight (Estrada et al., 2024a). Pre-farrow adjusted sow weight was estimated as total pigs born × 0.039 × days until farrowing from time of weighing + pre-farrow weight (Estrada et al., 2024a). Equations were adapted from historical estimations of sow weight and conceptus weight (Mallmann et al., 2018; Thomas et al., 2018).

Adequate colostrum intake was ensured for all pigs by following the Carthage System’s SOP for day 1 pig care. During and shortly after parturition, pigs were placed at the underline of the sow to encourage nursing. Pigs that were suspected of not nursing were placed under a heat lamp or in a warming box if chilled. A split-suckling approach was used where pigs that had been observed nursing were moved to a warming box to ensure all pigs had access to colostrum. Those that were not observed nursing were marked and placed at the underline of the sow. Pigs were not away from the sow for more than 45 min.

A modified management change to reduce exposure to bacteria to eliminate losses from PRRSv (McREBEL) program was used as the cross-foster management program. Piglet movements (i.e., cross-fostering) were permitted only for litters over functional teat capacity and litters of only 1 or 2 total pigs born alive. Morbid piglets (pigs that experienced excess weight loss or became injured) were not removed from the litter or moved to another sow. Any piglet that would have been categorized as a morbidity was humanely euthanized and counted as a mortality. Starting litter inventory number was calculated by subtracting the number of pigs that died prior to 48 hours of farrowing from the number of pigs born alive and adding or subtracting pigs that had to be cross-fostered. The weight of the pigs that died within 48 hours of being born was subtracted from the litter birth weight and weights of pigs cross-fostered were added or subtracted to determine a starting litter weight which was used to calculate litter average daily gain. Litter gain was calculated by subtracting the starting litter weight from the litter weaning weight. Litter ADG was calculated by dividing litter gain by the number of days that elapsed from the starting weight and weaning weight. The date, weight, and reason for mortality were recorded for all pigs throughout lactation. Litters that started on teats and then needed to be moved to another sow because the sow could not produce enough milk or died during the lactation period were classified as nurseoffs. The number of litters started on test and were weaned are listed in Tables 6 and 7, respectively.

**Table 6.** Effects of increasing soybean meal concentration fed during lactation on farrowing performance^1^

	Soybean meal (SBM) inclusion Level, %					
Item	20	25	30	35	SEM	*P*—value
Sows farrowed, n	232	244	239	245		
Total pigs born, n	15.66	15.89	16.00	16.42	0.26	0.21
Pigs born alive, n	11.88	12.31	12.08	12.39	0.23	0.37
Pigs born alive, %	76.98	77.84	75.99	75.91	1.63	0.81
Stillbirths, n	1.45^a^	1.53^ab^	1.80^c^	1.68^bc^	0.09	0.02
Stillbirths, %	9.47	10.08	11.47	10.67	0.70	0.25
Mummies, n	2.33	2.06	2.14	2.35	0.30	0.85
Mummies, %	14.46	12.80	14.24	14.08	1.10	0.51
Pre-cross foster mortalities^2^, n	1.16	1.35	1.28	1.23	0.08	0.30
Pre-cross foster mortalities^2^, %	8.67	10.05	9.77	9.11	0.71	0.48
Starting litter inventory, n	11.38	12.05	11.59	11.69	0.23	0.21
Nurseoffs^3^, n	0.37	0.39	0.19	0.40	0.14	0.83
Nurseoffs^3^, %	2.45	2.76	1.36	2.30	0.93	0.70
Post-cross foster mortalities^4^, n	1.32	1.25	1.10	1.32	0.08	0.31
Post-cross foster mortalities^4^, %	12.93	11.30	9.99	11.68	0.88	0.10

^a,b,c,d^Treatment means within a row that do not share a common letter significantly differ (*P* < 0.05).

^1^A total of 960 sows were used from farrowing until weaning. Sows were weighed, blocked by parity and randomly assigned to 1 of 4 diets with the factor being soybean meal level (20%, 25%, 30%, 35%). Movement from gestation to farrowing occurred at a target of 112 d of gestation. Weaning occurred at d 14 to 24 of lactation.

^2^Pre-cross foster mortalities—mortalities that occurred before the 48-h cross fostering period.

^3^Nurseoffs—piglets removed after 48 h that needed to be moved to another sow because the sow could not produce enough milk or died during the lactation period.

^4^Post-cross foster mortalities—mortalities that occurred after the 48-h cross fostering period. A modified management change to reduce exposure to bacteria to eliminate losses from PRRS (McREBEL) program was used as the cross-foster management program. Piglet movements (i.e., cross-fostering) were permitted only for litters over teat compacity and litters of only 1 or 2 total pigs born alive. Morbid piglets (pigs that experienced excess weight loss or became injured) were not removed from the litter or moved to another sow. Any piglet that would have been categorized as a morbidity was humanely euthanized and counted as a mortality.

**Table 7.** Effects of increasing soybean meal concentration fed during lactation on litter and individual pig performance^1^

	Soybean meal (SBM) inclusion level, %					
Item	20	25	30	35	SEM	*P*—value
Litters weaned, n	209	219	222	227		
Litter performance						
Litter birth weight, kg	16.82	17.65	16.98	17.44	0.44	0.47
Litter starting weight^2^, kg	16.74^a^	18.00^b^	16.86^a^	17.21^a^	0.34	0.03
Litter weaning weight, kg	66.98	70.39	70.13	69.03	1.33	0.21
Litter average daily gain, kg/d	2.57	2.65	2.67	2.63	0.05	0.47
Total litter gain^3^, kg	52.61	54.86	55.29	54.20	1.05	0.24
Pigs weaned, n	10.05	10.38	10.12	10.12	0.22	0.71
Individual piglet performance						
Coefficient of variation of weight of total pigs born, %	30.02	28.86	30.60	30.91	1.11	0.54
Average birth weight, kg	1.45	1.47	1.43	1.45	0.02	0.40
Coefficient of variation of birth weight of pigs born alive, %	20.51	20.52	20.22	20.63	0.49	0.94
Average starting weight^2^, kg	1.49	1.47	1.51	1.49	0.02	0.22
Average weaning weight, kg	6.83	6.78	6.86	6.83	0.08	0.89
Coefficient of variation of individual pig weaning weight, %	16.19	15.43	16.23	16.40	0.34	0.19
Average daily piglet gain, g/d	262.21	256.04	261.35	260.26	3.22	0.49

^a,b,c,d^Treatment means within a row that do not share a common letter significantly differ (*P* < 0.05).

^1^A total of 960 sows were used from farrowing until weaning. Sows were weighed, blocked by parity and randomly assigned to 1 of 4 diets with the factor being soybean meal level (20%, 25%, 30%, 35%). Movement from gestation to farrowing occurred at a target of 112 d of gestation. Weaning occurred at d 16 to 25 of lactation.

^2^Litter starting weight = weight at 48-h cross-fostering period.

^3^Total litter gain = litter weaning weight—litter starting weight.

Individual pig weaning weights were recorded using RFID tags (LeeO, Prairie Systems, Spencer, IA) and used to calculate litter weaning weight. Sows were weighed and measured with a caliper at the last rib (Knauer and Baitinger, 2015) at weaning. Sow weight change was calculated by subtracting the estimated sow weight after farrowing from the sow weight at weaning. Lactation feed efficiency was calculated by subtracting litter starting weight from litter weaning weight and dividing by total sow lactation feed intake (Estrada et al., 2024b).

### Statistical Analysis

Analyses of all continuous variables were conducted using the Mixed procedure of SAS version 9.4 (SAS, Cary, North Carolina). Sows were randomly assigned to treatment within parity group and blocked by replication within wave (groups of approximately 250 sows evaluated at a time). The only fixed effect in the model was soybean meal inclusion rate. Block nested in wave was included as random variable.

Discrete count variables (i.e., parity, total pigs born, pigs born alive, stillbirths, mummies, mortalities before cross-fostering, mortalities after cross-fostering, nurseoff piglets, and number of pigs weaned) were analyzed using the Glimmix procedure of SAS as a Poisson distribution using the log link option or a negative binomial distribution in the case of overdispersion.

Proportional data (i.e., percentage of pigs born alive, percentage of stillbirths, percentage of mummies, percentage of mortalities before cross foster, percentage of nurseoff piglets, and percentage of mortalities after cross foster) were analyzed using the Glimmix procedure of SAS as a beta-binomial distribution using the logit link option and a random intercept variable.

A Kenward-Roger degrees of freedom approximation was used to compute the denominator degrees of freedom for all variables due to differences in observations among treatments. Means separation was determined using the PDIFF option in both the Mixed and Glimmix procedures of SAS. Least squares mean differences for main effects were considered statistically different from 0 at *P* < 0.05 and tendencies at *P* < 0.10. Uncertainty of the estimates of least squares means was expressed as the maximum standard error among the 4 soybean meal inclusion levels.

## RESULTS

### Summary Statistics of Sow and Pig Performance

Body condition score into farrowing was 3.02 units (Table 3). Body condition score at weaning was 2.92 units. Sow weight into farrowing was 256.56 kg, and sow weight at weaning was 233.38 kg. Estimated sow weight after farrowing was 232.63 kg. Lactation daily feed intake was 5.99 kg, and total lactation feed intake was 131.36 kg. There were 16 total pigs born, 12.17 pigs born alive, 1.62 stillborn, and 2.22 mummies per litter. The starting litter inventory was 11.70 pigs and 10.42 pigs were weaned. Total litter birth weight (to include all live pigs, stillbirths, and mummies) was 17.23 kg, litter starting weight was 17.20 kg, and litter wean weight was 69.15 kg. Litter gain was 54.25 kg. Lactation G:F was 0.393 units. The average birth weight of each pig was 1.45 kg. The average starting weight of each pig was 1.49 kg. The average weaning weight of each pig was 6.82 kg. The average daily pig gain was 259.93 g/d.

**Table 3.** Population summary statistics of sow and pig performance

Item	Observations	Mean	SD	CV(%)	Median	Minimum	Maximum
Body condition score (BCS) into farrowing	960	3.02	0.25	8.26	3.00	2.00	4.00
Body condition score (BCS) at weaning	851	2.92	0.41	14.11	3.00	0.00	4.00
Sow weight into farrowing, kg	960	256.56	27.82	10.85	258.96	180.05	350.11
Sow weight at weaning, kg	855	233.38	34.73	14.88	237.19	131.97	331.97
Estimated sow weight after farrowing^1^, kg	957	232.63	27.88	11.98	235.37	160.09	338.78
Lactation daily feed intake, kg	850	5.99	1.08	18.10	6.02	2.10	9.52
Total lactation feed intake, kg	850	131.36	24.71	18.81	131.58	48.23	204.58
Total born, n	959	16.00	4.32	27.01	17.00	1.00	27.00
Born alive, n	959	12.17	4.97	40.87	13.00	0.00	26.00
Stillborn, n	959	1.62	1.77	109.50	1.00	0.00	11.00
Mummies, n	959	2.22	3.98	179.59	0.00	0.00	20.00
Starting litter inventory, n	895	11.70	3.37	28.81	12.00	2.00	19.00
Pigs weaned, n	851	10.42	3.08	29.56	11.00	1.00	17.00
Litter birth weight, kg	958	17.23	6.68	38.80	18.28	0.00	32.06
Litter starting weight^2^, kg	896	17.20	5.12	29.75	17.59	0.00	30.93
Litter wean weight^3^, kg	851	69.15	19.07	27.58	70.92	11.07	118.74
Litter gain^4^, kg	851	54.25	15.09	27.81	55.18	7.61	96.68
Lactation G:F^5^,	848	0.393	0.111	28.247	0.390	0.060	0.900
Average birth weight of pigs, kg	942	1.45	0.26	17.72	1.43	0.82	2.62
Average starting weight of pigs^2^, kg	894	1.49	0.22	15.04	1.48	0.93	2.33
Average wean weight^3^, kg	850	6.82	1.22	17.88	6.72	3.94	11.15
Average daily pig gain, g/d	850	259.93	46.10	17.74	254.82	141.52	431.34

^1^Estimated sow weight into farrowing = pre-farrow adjusted weight—conceptus weight. Pre-farrow adjusted weight = total born × 0.039 × days until farrowing from time of weighing + pre-farrowing weight. Sow weight into farrowing was the weight of the sow when placed in the farrowing crate at approximately d 112 of gestation. Conceptus weight = 0.137 + 1.329 × total pigs born × average pig birth weight (Estrada et al., 2024b, doi:10.1093/jas/skae247).

^2^Litter starting weight = weight after 48 hours cross foster period.

^3^Pig weaning weights were obtained an average of 1.35 d prior to weaning, therefore, lactation length is from date of farrowing until date that weaning weights were obtained.

^4^Litter gain = litter weaning weight—litter starting weight.

^5^Lactation gain-to-feed (G:F) was calculated as (litter weaning weight—litter starting weight) ÷ total sow lactation feed intake (Estrada et al., 2024b, doi:10.1093/jas/skae247).

### Sow Measurements and Feed Consumption

Parity was not different among treatments (Table 4, *P* = 0.51). Caliper at entry into farrowing room and at weaning were not different (*P* ≥ 0.12) among treatments. Body condition scores at entry into farrowing room were not different (*P* = 0.32) among treatments, however BCS at weaning was at least 0.09 units greater (*P* ≤ 0.03) in sows fed 20% SBM compared to all other treatments. Sow weight into farrowing, estimated sow weight after farrowing, and conceptus weight were not different (*P *≥ 0.25) among treatments. Sow weight at weaning and sow weight change during lactation were not different (*P* ≥ 0.17) among treatments. However, sow weights at weaning numerically followed the same trend as body condition scores at weaning.

**Table 4.** Effects of increasing soybean meal concentration fed during lactation on sow weight and body composition^1^

	Soybean meal (SBM) inclusion level, %					
Item	20	25	30	35	SEM	*P*—value
Sows, n	232	244	239	245		
Parity	3.53	3.36	3.62	3.50	0.12	0.51
Caliper at entry into farrowing	10.43	10.54	10.52	10.48	0.11	0.87
Caliper at weaning	9.81	9.41	9.43	9.40	0.16	0.12
Body condition score (BCS) into farrowing	3.03	3.04	3.01	3.00	0.02	0.32
Body condition score (BCS) at weaning	2.99^b^	2.90^a^	2.89^a^	2.89^a^	0.03	0.03
Sow weight into farrowing, kg	257.33	256.59	256.12	257.39	1.75	0.46
Estimated sow weight after farrowing^2^, kg	233.97	232.60	232.17	233.01	1.75	0.39
Conceptus weight^3^, kg	26.32	27.34	26.96	27.68	0.52	0.25
Sow weight at weaning, kg	236.81	233.23	233.37	233.79	2.24	0.17
^4^Sow weight change during lactation, kg	3.55	1.71	2.32	1.34	1.06	0.33
Sow weight change during lactation, %	1.39	0.54	0.78	0.36	0.47	0.30

^a,b,c,d^Treatment means within a row that do not share a common letter significantly differ (*P* < 0.05).

^1^A total of 960 sows were used from farrowing until weaning. Sows were weighed, blocked by parity and randomly assigned to 1 of 4 diets with the factor being soybean meal level (20%, 25%, 30%, 35%). Movement from gestation to farrowing occurred at a target of 112 d of gestation. Weaning occurred at d 14 to 24 of lactation.

^2^Estimated sow weight after farrowing = Adjusted weight before farrowing—conceptus weight. Adjusted weight before farrowing = total piglets born × 0.039 × days until farrowing from time of last weighing + weight into farrowing (Estrada et al., 2024b, doi:10.1093/jas/skae247). Sow weight into farrowing was the weight of the sow when moved into the farrowing crate at approximately 112 d of gestation.

^3^Conceptus weight = 0.137 + 1.329 × total piglets born × average piglet birth weight (Estrada et al., 2024b, doi:10.1093/jas/skae247).

^4^Sow weight change = weight at weaning—estimated sow weight after farrowing.

Sows fed 20% SBM in the diet consumed at least 1.74 g/d more (Table 5, *P* ≤ 0.05) of SID lysine compared to all other treatments. Sows fed 20% SBM in the diet consumed at least 0.57 Mcal/d more (*P* ≤ 0.05) daily metabolizable energy compared to all other treatments. Loading to weaning total intake for sows fed 20% SBM was increased (*P* < 0.01) by 6.12 kg and 5.98 kg compared to sows fed 30% SBM and 35% SBM respectively. Loading to weaning total intake for sows fed 25% SBM was marginally decreased by 3.65 kg compared to those fed 20% SBM (*P = *0.07) but was not different (*P *≥ 0.21) from all other treatments. Loading to weaning ADFI for sows fed 20% SBM was at least 0.15 kg greater (*P* ≤ 0.05) than all other treatments. Lactation total feed intake for sows fed 20% SBM was increased (*P* ≤ 0.02) by 4.76 kg and 5.18 kg compared to sows fed 30% SBM and 35% SBM, respectively. Lactation total feed intake for sows fed 25% SBM was not different (*P *≥ 0.12) from all other treatments. Lactation ADFI for sows fed 20% SBM was increased (*P *≤ 0.05) by at least 0.17 kg compared to all other treatments. Lactation G:F was decreased (*P *< 0.01) by at least 0.03 units in sows fed 20% SBM compared to all other treatments.

**Table 5.** Effects of increasing soybean meal concentration fed during lactation on sow total feed intake and average daily feed intake^1^

	Soybean meal (SBM) inclusion level, %					
Item	20	25	30	35	SEM	*P*—value
Sows, n	232	244	239	245		
Daily SID lysine intake, g/d	63.72^b^	61.98^a^	60.65^a^	60.82^a^	0.75	< 0.01
Daily metabolizable energy intake, Mcal/d	20.73^b^	20.16^a^	19.73^a^	19.79^a^	0.25	< 0.01
Loading to weaning total intake^2^, kg	144.73^b^	141.08^ab^	138.61^a^	138.75^a^	1.73	0.01
Loading to weaning ADFI^2^, kg	5.45^b^	5.30^a^	5.21^a^	5.23^a^	0.06	0.01
Lactation total intake, kg	134.46^b^	132.33^ab^	129.70^a^	129.28^a^	1.70	0.03
Lactation ADFI, kg	6.19^b^	6.02^a^	5.89^a^	5.91^a^	0.07	< 0.01
Lactation G:F^3^	0.368^a^	0.397^b^	0.408^b^	0.397^b^	0.01	< 0.001

^a,b,c,d^Treatment means within a row that do not share a common letter significantly differ (*P* < 0.05).

^1^A total of 960 sows were used from farrowing until weaning. Sows were weighed, blocked by parity and randomly assigned to 1 of 4 diets with the factor being soybean meal level (20%, 25%, 30%, 35%). Movement from gestation to farrowing occurred at a target of 112 d of gestation. Weaning occurred at d 14 to 24 of lactation.

^2^Time of movement from gestation to farrowing room through weaning of the piglets.

^3^Lactation G:F = (litter weaning weight—litter starting weight) ÷ sow total lactation feed intake.

### Farrowing, Litter, and Individual Pig Performance

The number of total pigs born was not different (Table 6, *P *= 0.21) among treatments. The number and percentage of pigs born alive were not different (*P* ≥ 0.37) among treatments. The number of stillbirths for sows fed 20% SBM was reduced (*P* ≤ 0.04) by 0.35 pigs and 0.23 pigs compared to sows fed 30% and 35% SBM. Sows fed 25% SBM had 0.16 fewer (*P* = 0.02) stillbirths compared to sows fed 30% SBM but stillbirths were not different (*P* ≥ 0.18) from sows fed 20% SBM or 35% SBM. The percentage of stillbirths were not different (*P *= 0.25) among treatments. The number and percentage of mummies was not different (*P* ≥ 0.51) among treatments. The number and percentage of pre-cross foster mortalities and starting litter inventory were not different (*P* ≥ 0.21) among treatments. The number and percentage of nurseoff pigs was not different among treatment groups (*P* ≥ 0.65). The number and percentage of post-cross-fostered mortalities were not different (*P* ≥ 0.10) among treatments.

Litter birth weight was not different (Table 7, *P *≥ 0.47) among treatments. Litter starting weight for sows fed 25% SBM at least 1.74 kg heavier compared to all other treatment groups (*P* ≤ 0.05). Litter starting weight had a tendency to increase (*P *= 0.09) in sows fed 25% SBM compared to sows fed 35% SBM. Litter weaning weight, litter ADG, and total litter gain were not different (*P* ≥ 0.21) among treatments. The number of pigs weaned was not different (*P* = 0.71) among treatments.

Total born weight coefficient of variation (CV) was not different (*P* = 0.54) among treatments. Average birth weight was not different (*P* = 0.40) among treatments. Birth weight CV was not different (*P* = 0.94) among treatments. Average starting weight, average weaning weight, and weaning weight CV were not different (*P* ≥ 0.19) among treatments. Average daily piglet gain was not different (*P* = 0.49) among treatments.

## DISCUSSION

Porcine reproductive and respiratory syndrome virus is a single-stranded positive-sense RNA virus (Cho and Dee, 2006). It causes reproductive failures such as stillborn piglets, mummies, and late term abortions in sows, and increases mortality and hinders growth performance in pre-weaned pigs (Tousignant et al., 2015). Estrada et al. (2024b) used the same population of sows prior to being endemically infected with PRRSv. This study was conducted in the same season a year prior to the current study and was used as a baseline for statistical comparison (Suplementary Fig 1A-1L). Piglet and sow performance were significantly impacted in the current study compared to Estrada et al. (2024b).

The number of total born pigs per litter decreased from 16.68 to 16.00 (*P < *0.01) pre-and post-outbreak. This corresponded to a decrease in the absolute number of pigs born alive per litter of 2.66 (*P *<* *0.01) or 12.88% (*P *<* *0.01) as a percentage of total born. The percentage of stillborn pigs per litter significantly increased by 1.29 (*P *< 0.01) while the increase in the number of stillborn pigs was marginally significant (0.14, *P *= 0.06). The number of mummified pigs markedly increased by 1.84 per litter (*P *< 0.01) or 11.10% relative to total born (*P* < 0.01). The number of fallouts significantly decreased (0.27, *P* < 0.01); however, this is largely attributed to the large decrease in the number of pigs started after cross-fostering (2.79, *P* < 0.01) since it was not significant as a percentage of pigs started (*P* = 0.50). The resulting number weaned was decreased by 1.87 pigs post outbreak (*P < *0.01).

Linhares et al. (2014) found the total losses (i.e., the number of pigs not weaned following whole-herd exposure to PRRSV) was 2,217 pigs/1,000 sows (95% CI: 2,052 to 3,278). Additional work conducted by Trevisan et al., (2022) found the total losses to be 2,233 pigs/1,000 sows (95% CI: 1,412 to 3,056). In the current study, the whole herd was first vaccinated with a commercial PRRSv modified live vaccine followed by whole-herd exposure between 3 and 4 wk later. The total loss measured in the current study was estimated to be 1,870 pigs/1,000 sows (95% CI: 1,514 to 2,232). While not a precise comparison, the total losses measured in this trial attributed to PRRSv infection are relatively consistent with previous research. Given the total losses, the increase in mummified pigs, and the persistently strong Cq values measured in this study, it is reasonable to conclude that the PRRSv outbreak likely had a significant, negative impact on the herd.

Soybean meal provides high levels of crude protein (44% to 48%) and energy (net energy 2,233 kcal/kg) in the swine diet (Gaffield et al., 2024), making SBM a high-quality ingredient included in many swine diets. Numerous studies have investigated increasing the inclusion of SBM in swine diets. However, many of these studies simultaneously increased Lys levels along with SBM. As an example, Estrada et al (2024b) reported increasing SID Lys from approximately 15% to 26% by increasing SBM in the lactation diet fed to healthy sows had little impact on the net litter performance. Greiner et al. (2020) completed multiple experiments with increasing levels of SBM in the lactation diet of healthy sows. They reported that, as SBM increased from 25% to 40%, piglet ADG and daily litter gain linearly improved (Greiner et al., 2020). However, this result was not consistent across all experiments. Swanstrom et al. (2023) fed 3 levels of SBM ranging from approximately 26% to 48% to healthy grow-finish pigs and reported no differences in overall growth performance. It is important to note again that in the studies mentioned, Lys levels in the diets were also increasing along with SBM which could be a reason for differences in responses compared to the current study where Lys levels were held constant across dietary treatments. Wileman et al. (2024) fed gilts a corn-soy diet supplemented with synthetic amino acids and then fed high levels of SBM to replace all other synthetic amino acids. Wileman et al. (2024) reported no differences in growth performance of the growing pigs regardless of the SBM inclusion in the diets. Similarly, Gourley et al. (2020) found that keeping Lys levels in constant resulted in no differences in litter size, litter weight, or litter wean weight as SBM increased from 25% to 35%. However, it is important to acknowledge the important limitation that amino acids were not profiled in either the current study or in Gourley et al. (2020).

When Lys level is constant regardless of SBM, there is no impact on the growth performance of the pigs in healthy conditions. Because there appears to be a greater response to SBM when pigs are health-challenged, it was hypothesized that the benefits of feeding SBM are not as prevalent in a pig unless an immune challenge or infection is present. It was unclear whether the beneficial effects of soybean meal was a result of increased crude protein, other bioactive compounds, or a combination of both (Boyd et al., 2010). There are a multitude of bioactive compounds in soybean meal that potentially can mitigate immune stress and support performance such as select carbohydrates, polyphenols (including isoflavones), and saponins (White et al., 2024).

Therefore, it was expected that as SBM inclusion levels increased in the diets fed to sows that were endemically infected with PRRSv, sow and litter performance would improve. Under the conditions of this study, this was not the result. While lactation feed efficiency did improve above 20%, the lowest SBM inclusion level resulted in improved lactation feed intake and better body condition at weaning of the sows consistent with previous research (Estrada et al., 2024a) Soy isoflavones when fed in a purified form can reduce mortality of growing pigs (Smith et al., 2020) and improve growth performance of nursery pigs (Rochell et al., 2015). Growing pigs infected with PRRSv using a 1.0 × 10^5^ 50% tissue culture infective dose of PRRS virus (strain NADC20, courtesy of Dr. Federico Zuckermann, University of Illinois, Urbana, IL) and fed 1,635 mg/kg isoflavones (1,396 mg/d—4,355.64 mg/d) reduced pathogen-associated mortality by approximately 50% compared to pigs fed a diet practically devoid of isoflavones (Smith et al., 2020). However, there was little impact of isoflavone level on overall growth performance from weaning to market (Smith et al., 2020). On the other hand, Rochell et al. (2015) reported increasing the SBM level from 17.5% to 29% (700 mg/kg vs 1246 mg/kg isoflavones) tended to improve growth performance in the nursery phase for pigs infected with PRRSv using a 1 × 10^5^ 50% tissue culture infective dose of PRRSV (P-129 isolate, Purdue University, West Lafayette, IN). Nursery pigs consumed approximately 414 mg/d isoflavones in the low SBM group compared to 770 mg/d in the high SBM group (Rochell et al., 2015). The current study had total isoflavones range from 739.50 mg/kg to 1165.00 mg/kg. Sows fed 20% SBM were consuming approximately 4,577.50 mg isoflavones a day, and sows fed 35% SBM were consuming approximately 6,885.15 mg isoflavones a day. These levels were naturally much greater than those in either Rochell et al. (2015) or Smith et al. (2020) as lactating sows objectively consume a much higher mass of feed.

In the case of Smith et al. (2020) and Rochell et al. (2015), isoflavones were directly fed to the nursery and growing pigs to elicit a response whereas in the current trial, isoflavones were fed to the sow in hopes of achieving a growth response in the pre-weaned pig. Bryan et al. (2022) fed isoflavones (at a concentration of 1,500 mg/kg)to gilts starting at d 70 of gestation. Again, isoflavone concentration in the current study ranged from 739.50 mg/kg to 1165.00 mg/kg and were fed to the sows from 112 of gestation through weaning. There was no information available on sow ADFI in the Bryan et al. (2022) experiment to estimate isoflavone daily intake. Bryan et al. (2022) reported no differences in birth or weaning weights of pigs compared to pigs born to sows fed a diet practically devoid of isoflavones. Bryan et al. (2022) also reported no differences in survival of pigs during the suckling period.

Still, there may be benefits to sow longevity and subsequent reproductive performance. Sows that were fed 20% SBM in the diet maintained better body condition because of better lactation feed intake. The concentration of isoflavones in the diet increased with increasing dietary SBM. However, it is unclear whether isoflavones exhibit a protective effect when provided through SBM in PRRSv challenged sows. It is worth emphasizing that there are many bioactive compounds in soybean meal in addition to isoflavones. Further testing is necessary to understand the effects of the level of SBM fed to endemically infected PRRSv positive sows and whether this protection has a lasting effect on subsequent performance and sow retention.

## CONCLUSION

Overall, feeding sows endemically infected with PRRSv increasing levels of SBM did not result in a mortality reduction or improve litter performance during the current lactation; however, sows fed higher levels of soybean meal were more feed efficient because daily feed intake reduced as SBM increased. Sows fed a diet including 20% SBM had greater feed intake and improved body condition at weaning which may position those sows to have improved subsequent performance and longevity within the herd. Future research should investigate the longevity and subsequent performance of the sows, as well as the lifetime growth performance of the offspring to conclude if reducing SBM in the lactation diet better prepares a sow for her next lactation.

## Supplementary Material

txaf054_suppl_Supplementary_Figure_S1

## References

[CIT0001] AOAC. 1996. Official Methods of Analysis of AOAC International. 15th ed. Arlington, VA: Association of Official Analytical Chemists.

[CIT0002] AOAC. 2005. Official Methods of Analysis of AOAC International. 18th ed. Gaithersburg, MD: AOAC Int.

[CIT0003] AOAC. 2006. Official Methods of Analysis of AOAC International. 18th ed. Gaithersburg, MD: AOAC Int.

[CIT0004] AOAC. 2007. Official Methods of Analysis of AOAC International. 18th ed. Gaithersburg, MD: AOAC Int.

[CIT0005] AOAC. 2012. Determination of Ash in Animal Feed: AOAC Official Method 942.05 Revisited. Gaithersburg, MD: AOAC Int. 10.5740/jaoacint.12-129 23175971

[CIT0006] Boyd, R. D., M. E. Johnston, and C. Zier-rush. 2010. Soybean meal level modulates the adverse effect of high immune stress on growth and feed efficiency in growing pigs. Proc. MN Nutr. Conf. 71:167–174.

[CIT0007] Bryan, E. E., X. Chen, B. N. Smith, R. N. Dilger, and A. C. Dilger. 2022. Maternal immune activation and dietary soy isoflavone supplementation influence pig immune function but not muscle fiber formation. J. Anim. Sci. 100:skac134. doi: https://doi.org/10.1093/jas/skac134 35426431 PMC9155173

[CIT0008] Cho, J. G. and S. A. Dee. 2006. Porcine reproductive and respiratory syndrome virus. Theriogenology. 66:655-662. doi: https://doi.org/10.1016/j.theriogenology.2006.04.024 16730057

[CIT0009] Estrada, J., D. C. Johnson, K. L. Kyle, J. Perez, E. Parr, M. W. Welch, C. Neill, B. A. Peterson, and D. D. Boler. 2024a. Characterizing sow feed intake during lactation to explain litter and subsequent farrowing performance. J. Anim. Sci. 102:skae093. doi: https://doi.org/10.1093/jas/skae093 38558022 PMC11044703

[CIT0010] Estrada, J., J. G. Perez, K. L. Kyle, E. Parr, M. W. Welch, D. C. Johnson, C. Neill, and D. D. Boler. 2024b. Effects of increasing standard ileal digestibility lysine and metabolizable energy levels in lactation diets fed to young and mature sows. J. Anim. Sci. 103:skae247. doi: https://doi.org/10.1093/jas/skae247 PMC11979742 39180183

[CIT0011] Gaffield, K. N., R. D. Goodband, J. M. DeRouchey, M. D. Tokach, J. C. Woodworth, G. Denny, and J. T. Gebhardt. 2024. A review of soybean processing by-products and their use in swine and poultry diets. Transl. Anim. Sci. 8:txae063. doi: https://doi.org/10.1093/tas/txae063 38689757 PMC11059257

[CIT0013] Gourley, K. M., J. C. Woodworth, J. M. DeRouchey, M. D. Tokach, S. S. Dritz, and R. D. Goodband. 2020. Effects of soybean meal concentration in lactating sow diets on sow and litter performance and blood criteria. Transl. Anim. Sci. 4(2). txaa037. doi: https://doi.org/10.1093/tas/txaa037 32705034 PMC7201160

[CIT0012] Greiner, L., P. Srichana, J. L. Usry, C. Neill, G. L. Allee, J. Connor, K. J. Touchette, and C. D. Knight. 2020. Lysine (protein) requirements in lactating sows. Transl. Anim. Sci. txaa072. doi: https://doi.org/10.1093/tas/txaa072 4 32705067 PMC7288739

[CIT0014] Havas, K. A., L. Brands, R. Cochrane, G. D. Spronk, J. Nerem, and S. A Dee. 2023. An assessment of enhanced biosecurity interventions and their impact on porcine reproductive and respiratory syndrome virus outbreaks within a managed group of farrow-to-wean farms, 2020–2021. Front. Vet. Sci. 9:952383. doi: https://doi.org/10.3389/fvets.2022.952383 36713879 PMC9879578

[CIT0015] Hsieh, H. C., T. H. Kao, and B. H. Chen. 2004. A fast HPLC method for analysis of isoflavones in soybean. J. Liq. Chromatogr. Related Technol. 27:315-324. doi: https://doi.org/10.1081/jlc-120027102

[CIT0016] Knauer, M. T., and D. J. Baitinger. 2015. The sow body condition caliper. Appl. Eng. Agric. 31:175-178. doi: https://doi.org/10.13031/aea.31.10632

[CIT0017] Linhares, D. C., J. P. Cano, M. Torremorell, and R. B. Morrison. 2014. Comparison of time to PRRSv-stability and production losses between two exposure programs to control PRRSv in sow herds. Prev Vet Med 116(1-2):111-9 doi: https://doi.org/10.1016/j.prevetmed.2014.05.010 24931129

[CIT0018] Mallmann, A. L., G. D. S. Oliveira, J. Z. Rampi, F. B. Betiolo, D. P. Fagundes, J. E. G. Faccin, I. Andretta, R. D. R. Ulguim, A. P. G. Mellagi, and F. P. Bortolozzo. 2018. Proposal of equations for predicting post-farrowing sow weight. Acta Sci. Vet. 46:8. doi: https://doi.org/10.22456/1679-9216.83867

[CIT0019] Nan, Y., C. Wu, G. Gu, W. Sun, Y. J. Zhang, and E. M. Zhou. 2017. Improved vaccine against PRRSV: current progress and future perspective. Front. Microbiol. 8:1635. doi: https://doi.org/10.3389/fmicb.2017.01635 28894443 PMC5581347

[CIT0020] Nutrient Requirements of Swine (NRC). 2012. Nutrient Requirements of Swine. 11th rev. ed. Washington, DC: National Academies Press.

[CIT0021] Osemeke, O. H., G. De Souza E Silva, C. Corzo, M. Kikuti, Y. Xiaomei, D. Correia Lima Linhares, and D. J. Holtkamp. 2024. Updating the productivity and economics costs of PRRSV in the US. International Pig Veterinary Society Congress. June 4-7, 2024. Leipzig, Germany. [accessed August 15, 2024] https://www.ipvs2024.com

[CIT0022] Pedro Mil-Homens, M., S. Jayaraman, K. Rupasinghe, C. Wang, G. Trevisan, F. Dórea, C. L. Linhares, D. Holtkamp, and G. S. Silva. 2024. Early detection of PRRSV outbreaks in breeding herds by monitoring productivity and electronic sow feed data using univariate and multivariate statistical process control methods. Transbound. Emerg. Dis. 2024:9984148. doi: https://doi.org/10.1155/2024/9984148 40303118 PMC12017042

[CIT0023] Quezada-Fraide, E. A., C. G. Peñuelas-Rivas, F. S. Moysén-Albarrán, M. E. Trujillo-Ortega, and F. E. Martínez-Castañeda. 2021. Productive performance and costs of swine farms with different PRRS virus vaccination protocols. Rev. Mex. Cienc. Pecu. 12:205-216. doi: https://doi.org/10.22319/rmcp.v12i1.5377

[CIT0024] Rocha, G. C., R. D. Boyd, J. A. S. Almeida, Y. Liu, T. M. Che, R. N. Dilger, and J. E. Pettigrew. 2013. Soybean meal level in diets for pigs challenged with porcine reproductive and respiratory syndrome (PRRS) virus. J. Anim. Sci. 92(E-Suppl. 2):31.

[CIT0025] Rochell, S. J., L. S. Alexander, G. C. Rocha, W. G. Van Alstine, R. D. Boyd, J. E. Pettigrew, and R. N. Dilger., 2015. Effects of dietary soybean meal concentration on growth and immune response of pigs infected with porcine reproductive and respiratory syndrome virus. J. Anim. Sci. 93:2987-2997. doi: https://doi.org/10.2527/jas.2014-8462 26115285

[CIT0026] Smith, B. N., and R. N. Dilger. 2018. Immunomodulatory potential of dietary soybean-derived isoflavones and saponins in pigs. J. Anim. Sci. 96:1288–1304. doi: https://doi.org/10.1093/jas/sky036 29471443 PMC6140853

[CIT0027] Smith, B. N., A. Morris, M. L. Oelschlager, J. Connor, and R. N. Dilger. 2019. Effects of dietary soy isoflavones and soy protein source on response of weanling pigs to porcine reproductive and respiratory syndrome viral infection. J. Anim. Sci. 97:2989–3006. doi: https://doi.org/10.1093/jas/skz135 31011748 PMC6606490

[CIT0028] Smith, B. N., M. L. Oelschlager, M. S. Abdul Rasheed, and R. N. Dilger. 2020. Dietary soy isoflavones reduce pathogen-related mortality in growing pigs under porcine reproductive and respiratory syndrome viral challenge. J. Anim. Sci. 98:skaa024. doi: https://doi.org/10.1093/jas/skaa024 31960037 PMC7023622

[CIT0029] Swanstrom, J. A., D. C Humphrey, S. Elefson, K. A. Miller, S. Becker, C. S. Hagen, M. J. Nisley, L. L. Greiner, and N. K. Gabler. 2023. Effect of soybean meal inclusion on grow-finish pig performance and nitrogen balance. J. Anim. Sci. 101(Suppl.2):251-252. doi: https://doi.org/10.1093/jas/skad341.285

[CIT0030] Thomas, L. L., R. D. Goodband, M. D. Tokach, S. S. Dritz, J. C. Woodworth, and J. M. DeRouchey. 2018. Partitioning components of maternal growth to determine efficiency of feed use gestating sows. J. Anim. Sci. 96:4313-4326. doi: https://doi.org/10.1093/jas/sky219 29873742 PMC6162597

[CIT0031] Tousignant, S. J., A. M. Perez, J. F. Lowe, P. E. Yeske, and R. B. Morrison. 2015. Temporal and spatial dynamics of PRRSV infection in the United States. Am. J. Vet. Res. 76:70–76. doi: https://doi.org/10.2460/ajvr.76.1.70 25535663

[CIT0032] Trevisan, G., M. Zeller, G. Li, J. Zhang, P. Gauger, and D. C. L. Linhares. 2022. Implementing a user-friendly format to analyze PRRSV next-generation sequencing results and associating breeding herd production performance with number of PRRSV strains and recombination events. Transbound Emerg Dis 69(5):e2214-e2229. doi: https://doi.org/10.1111/tbed.14560 35416426 PMC9790532

[CIT0033] United Soybean Board. 2023. US soybean meal feed use by species. [accessed August 2024]. https://marketviewdb.unitedsoybean.org/dashboards/?bi=US_Meal_FeedUsebySpecies_Annual

[CIT0035] White, C. S., Froebel, L. E., and R. N. Dilger. 2024. A review on the effect of soy bioactive components on growth and health outcomes in pigs and broiler chickens. J. Anim. Sci 102:skae261. doi: https://doi.org/10.1093/jas/skae261 39234891 PMC11452720

[CIT0034] Wileman, C., Humphrey, D. C., Scoggin, K., Trabue, S., Bergstrom, J. R., Perez-Calvo, E. and Greiner, L.L.., 2024. 113 Evaluation of high soybean meal diets with inclusion of dietary enzymes on the effect of growth performance and greenhouse gas emissions. J. Anim. Sci. 102(Suppl.2):180-181. doi: https://doi.org/10.1093/jas/skae102.199

