# Supplementary figures and images for: Determining the appropriate soybean meal inclusion level in lactation diets for sows endemically infected with porcine reproductive and respiratory syndrome virus (PRRSv)
Source: Transl Anim Sci. 2025 May 4;9:txaf054. doi: 10.1093/tas/txaf054 (PMC12411667; doi:10.1093/tas/txaf054)

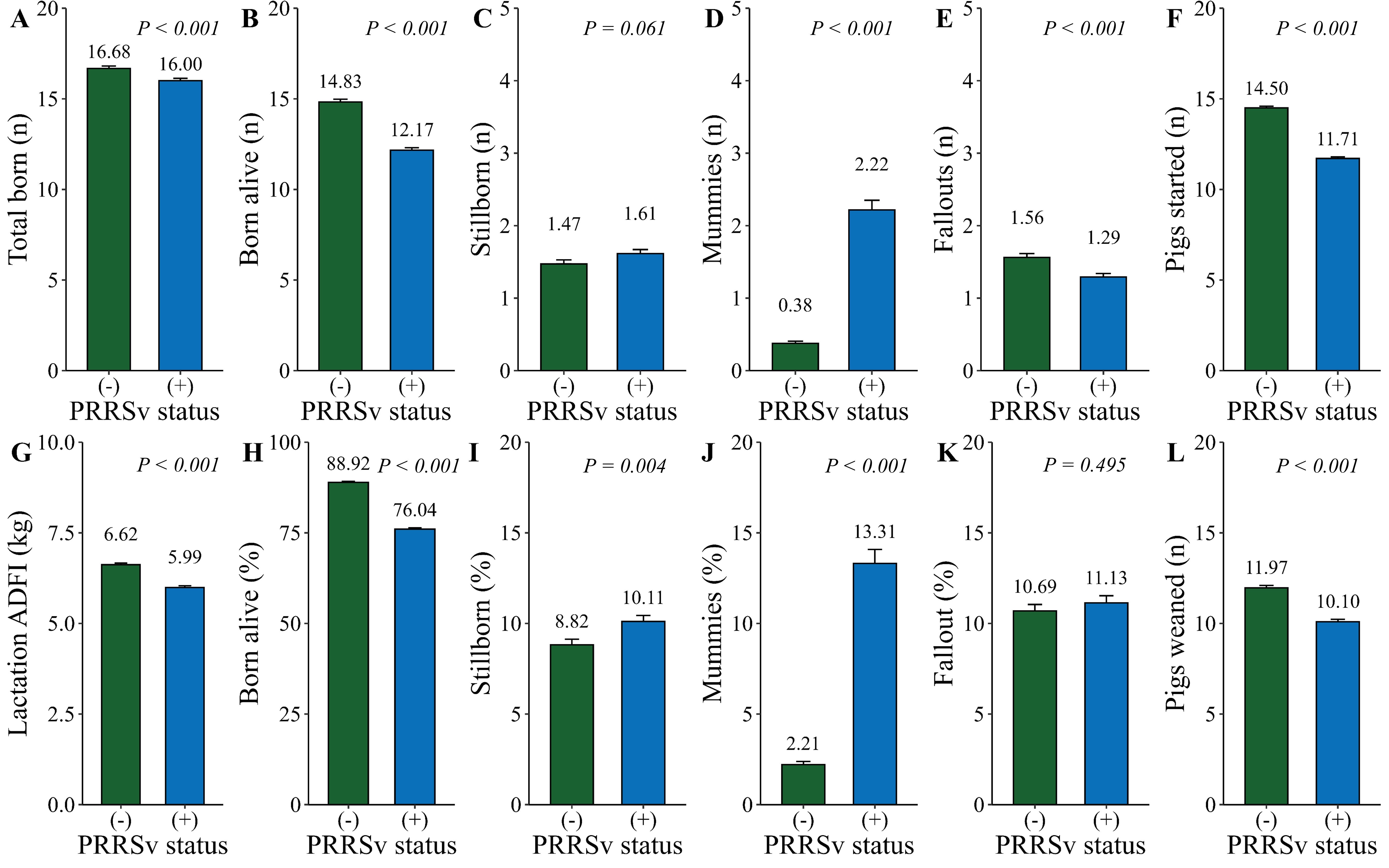

Supplement: txaf054_suppl_Supplementary_Figure_S1 [file txaf054_suppl_supplementary_figure_s1.jpeg]
